# Thriving in place: Multidimensional neighborhood typologies and cognitive function among U.S. older adults in the Health and Retirement Study

**DOI:** 10.1371/journal.pone.0344785

**Published:** 2026-03-12

**Authors:** Jiao Yu, Weidi Qin, Jiuzhou Wang, Eva Kahana

**Affiliations:** 1 School of Public Health, Yale University, New Haven, Connecticut, United States of America; 2 Sandra Rosenbaum School of Social Work, University of Wisconsin–Madison, Madison, Wisconsin, United States of America; 3 School of Public Health, University of Minnesota Twin Cities, Minneapolis, Minnesota, United States of America; 4 Department of Sociology, Case Western Reserve University, Cleveland, Ohio, United States of America; Ain Shams University, EGYPT

## Abstract

**Background:**

Neighborhood physical, social, and service environments are increasingly recognized as important contextual factors related to cognitive health; however, few studies have examined how these features collectively shape cognitive outcomes. This study aimed to classify neighborhood typologies based on a constellation of neighborhood features and to investigate their associations with cognitive function among U.S. older adults.

**Methods:**

We conducted a cross-sectional analysis of 6,480 participants from the 2016 wave of the Health and Retirement Study. To examine contemporaneous associations, neighborhood features were derived from the 2015 National Neighborhood Data Archive, including measures of neighborhood deprivation, service facilities, food access, healthcare facilities, and environmental hazards. Cognitive function was assessed using the 27-point Telephone Interview for Cognitive Status (TICS). The Partitioning Around Medoids (PAM) clustering algorithm was used to classify neighborhood typologies. Multilevel regression models were performed to examine the associations between neighborhood typologies and cognitive function, with individuals as the first level and census tracts as the second level.

**Results:**

Four neighborhood typologies were identified based on the elbow method and theoretical interpretability: (1) low deprivation, green neighborhood, (2) mid-SES, high hazard neighborhood, (3) high-amenity neighborhood, and (4) disadvantaged neighborhood. Regression results revealed significant disparities in cognitive function across neighborhood typologies. Compared to those living in disadvantaged neighborhoods, older residents in high-amenity neighborhoods characterized by extensive facilities and cognitively stimulating services demonstrated better cognitive function (*β =* 3.85*, 95% CI:* 1.23–6.47), after adjusting for individual-level characteristics.

**Conclusions:**

The identified neighborhood typologies reveal an unequal distribution of amenities and hazards, which may help explain considerable inequities in late-life cognitive health. Tailored community initiatives addressing amenity availability and environmental hazards could be pivotal in promoting cognitive health and supporting aging in place.

## Introduction

Cognitive decline represents a major health challenge in later life. In the United States, mild cognitive impairment affects approximately 22% of adults aged 65 years or older, and approximately 10% of this population lives with dementia [1]. There has been growing recognition that cognitive health disparities should be understood within specific social and environmental contexts, with neighborhood environments playing a particularly important role in shaping cognitive outcomes in later life. Compared to younger adults, older adults spend more time in their residential neighborhoods, and thus the importance of neighborhood environments may increase with age [2]. Neighborhoods are primary settings for social interaction, physical activity, and access to resources, thereby potentially facilitating or impeding health-promoting behaviors that can delay or accelerate cognitive aging. Extensive research has pointed to the connection between neighborhood characteristics and cognitive health outcomes [3–6]. However, much of this scholarship has examined isolated environmental features in relation to cognition, such as exposure to green space, food access, or neighborhood walkability [7–9]. While these findings offer valuable insights, few studies have examined how the totality of neighborhood contexts might influence cognitive health. That is, we know little about how combinations of neighborhood features collectively shape cognitive function. Understanding how these multidimensional and potentially interactive neighborhood features contribute to health is particularly important, as older adults’ experiences of place often reflect a dynamic interplay of physical, social, and structural conditions.

Neighborhood environments, as social determinants of health, play a significant role in shaping older adults’ cognitive health [4,5]. Empirical evidence suggests that socioeconomic deprivation, infrastructure, and amenities within one’s neighborhood can substantially influence older adults’ cognitive health. For example, cognitive decline is associated with living in neighborhoods with a high concentration of socioeconomically disadvantaged households [10,11], low walkability [9], and high levels of physical disorder [12]. Additionally, earlier evidence suggests that neighborhoods rich in diverse amenities (i.e., convenience stores, pharmacies, gyms, and recreation centers) may promote community-dwelling older adults’ mobility and participation in social activities [13,14], which in turn may delay cognitive decline and reduce the risk of cognitive impairment [15].

Neighborhood features, which serve as upstream factors, may either delay or accelerate cognitive impairment by shaping daily experiences and exposures related to physical activity, diet, pollutant exposure, and social engagement or isolation [16]. Amenity-rich neighborhoods featuring parks, recreation centers, or civic organizations may promote cognitive health by facilitating physical activity, social engagement, and mental stimulation, which are known to build cognitive reserve [4]. Conversely, denser urban neighborhoods with environmental stressors such as air pollution, noise, crowding, and physical disorder can elicit varying levels of chronic stress, which has been shown to have deleterious effects on brain structures critical for memory and learning [17,18]. Nevertheless, the existing literature has not yet provided the most plausible mechanisms or the precise directionality of associations between specific neighborhood features and cognitive outcomes. It is likely that the mechanisms vary depending on the particular feature under investigation. Moreover, rather than a single explanatory pathway, it is reasonable to assume that multiple mechanisms may operate interactively and reinforce one another to shape cognitive health outcomes [16].

Existing studies have largely examined neighborhood characteristics in isolation, focusing on individual dimensions such as socioeconomic disadvantage, amenities, polluting hazards, etc. [19–21]. While valuable, they fail to elucidate how the combination of different neighborhood features (i.e., both protective and risk factors) may collectively affect cognitive outcomes. Specifically, a neighborhood may contain a mixture of both protective factors that promote cognitive health (e.g., green space, libraries) and risk factors that accelerate cognitive decline (e.g., pollution, proximity to highways) [22]. For example, while a high level of concentrated disadvantage in a neighborhood may increase the risk of impaired cognitive function, it is also possible that older adults in socioeconomically disadvantaged neighborhoods experience stronger social ties with neighbors, thus buffering against health deterioration [23].

Additionally, neighborhoods constitute a critical milieu involving participants, physical space, activities, and interactions that collectively imbue a place with meaning [24]. These environments shape opportunities for engagement, access to resources, and exposure to risks, all of which accumulate in synergistic ways to influence well-being in later life [25]. Building on this perspective, the present study draws upon the ecological framework of aging to understand the complex interplay between individuals and their environments in shaping cognitive health [4,24,26]. The ecological theory of aging suggests that successful aging in place can be achieved through the optimization of person-environment fit [26]. That is, the balance between personal competence and environmental press plays an important role in late-life well-being. While risk factors within the environment may disrupt this balance by imposing excessive demands, protective factors may enhance person-environment fit by buffering against environmental stressors or by providing resources that support older adults’ capacities. Informed by this framework, we conceptualize neighborhood environments as upstream determinants of health that can constitute both opportunities and barriers to cognitive health. By examining neighborhood-cognition associations with consideration of multidimensional neighborhood risk and protective factors, our findings will provide an essential basis for developing tailored community interventions aimed at promoting cognitive resilience and supporting healthy aging in place.

Taken together, while prior research has established that individual neighborhood characteristics, such as socioeconomic disadvantage, environmental hazards, or neighborhood walkability, are associated with cognitive health, we know little about how the clustering of both risk and protective factors across multiple neighborhood dimensions collectively influences cognitive outcomes. Recent scholarship has advanced the framework of the exposome, which refers to the totality of environmental exposures that individuals encounter and their influences on health outcomes [27]. This holistic framework motivates our creation of multidimensional neighborhood typologies that provide a more comprehensive and realistic account of concurrent exposures within neighborhood contexts. Building on this premise, the present study aims to investigate multidimensional features of neighborhood environments in relation to cognitive function. We position our study as providing critical evidence that empirically derived neighborhood typologies are associated with meaningful variation in cognitive function among older adults, thereby informing future investigations examining neighborhood change, residential transitions, and cognitive aging trajectories. Using data from the nationally representative Health and Retirement Study (HRS) and applying a novel machine-learning clustering method, this research has two primary aims. First, we will identify neighborhood typologies based on neighborhood socioeconomic status, environmental hazards, and access to amenities and facilities. Second, we will examine how these neighborhood typologies are associated with cognitive function among community-dwelling older adults.

## Methods

### Data

Data were drawn from the Health and Retirement Study (HRS) and the National Neighborhood Data Archive. The HRS is a nationally representative longitudinal study of Americans aged 51 and older. Data have been collected biannually since 1992, and the sample is replenished every six years. Using a multistage probability sampling design, the HRS collected demographic, socioeconomic, and health data as well as biological subsamples for genomic and biomarker analyses [28]. The present study used data from participants who completed the 2016 wave core survey and provided salivary DNA samples with available genetic data collected between 2006 and 2012 [29].

Neighborhood data were obtained from the National Neighborhood Data Archive (NaNDA). NaNDA is a publicly available data repository containing nationwide contextual measures of the physical and social environments, for example, walkability, amenities, green space, and healthcare [30]. The HRS team geocoded respondents’ residential addresses to 2010 U.S. census tract boundaries, allowing us to link the HRS sample to tract-level contextual measures from NaNDA. We linked the 2016 HRS sample to the 2015 wave of NaNDA data at the census tract level to ensure appropriate temporal ordering; this approach captured neighborhood features one year before the collection of cognitive function measurements. There were 3,352 unique tracts in this study with an average of 2 persons per tract (range: 1–41).

We restricted the analysis to the 2016 wave of the Health and Retirement Study for several methodological and conceptual reasons. First, the 2016 cognitive assessment preceded the COVID-19 pandemic, thereby avoiding potential measurement artifacts associated with pandemic-related disruptions [31]. Second, using the 2016 wave minimized the temporal gap between neighborhood exposure measurement and cognitive outcome assessment, reducing potential mortality selection and attribution bias.

A total of 20,880 participants completed the 2016 wave of the HRS interview. We restricted our sample to community-dwelling adults aged 65 and older (n = 10,104). We excluded 780 participants with incomplete cognition information, 994 participants lacking genomic data, and 827 participants who moved between census tracts during 2015 and 2016. We further excluded 676 participants who lacked census tract information and thus could not be matched to the neighborhood data. Among the remaining participants, listwise deletion was applied to those with missing values in key covariates (n = 347). This resulted in a final analytic sample consisting of 6,480 community-dwelling older adults. Compared to participants included in the analytic sample, excluded individuals were more likely to be Black or Hispanic, have lower educational attainment, be non-working, be non-drinkers, be APOE ε4 carriers, and report greater difficulties with activities of daily living. Excluded participants were also more likely to reside in urban or suburban areas. No statistically significant differences were observed with respect to gender, smoking status, or number of chronic conditions. The sample selection process is shown in S1 Fig.

### Measures

#### 2.2.1. Cognitive function.

Cognitive function was assessed using an adapted version of the Telephone Interview for Cognitive Status (TICS), a cognitive scale with a maximum of 27 points [32]. Participants were asked a series of questions assessing (1) immediate and delayed word recall measuring memory (0–20 points); (2) a serial sevens subtraction test measuring working memory (0–5 points); and (3) a counting backwards test measuring mental processing speed (0–2 points). For respondents who were unable to participate, cognitive function was assessed through a proxy interview. In the present study, we excluded proxy respondents and only included cognitive scores from self-respondents. Cognitive function was measured as the sum of the component scores, with higher scores representing better cognitive function.

#### 2.2.2. Neighborhood features.

Neighborhood measures came from NaNDA (2015). Consistent with prior work and data availability, we used census tracts as proxies for neighborhoods [33]. Detailed methodology for the construction of these measures, including item classifications and buffer calculations is available from the NaNDA repository [34]. In this study, we included a comprehensive set of neighborhood features related to cognitive health [4]. These neighborhood features were classified into five categories: neighborhood deprivation, service facilities, food access, healthcare, and environmental hazards. A total of 21 individual measures were examined. All neighborhood measures were expressed as counts per 1,000 population within a census tract and top-coded at the 99^th^ percentile to reduce the influence of high-leverage points. Detailed information on neighborhood variables is presented in Table 1.

**Table 1:** Neighborhood-Level Contextual Measures, NaNDA (2015) ^a.^

Neighborhood Features	Indicators
Healthcare access	• Healthcare services including all ambulatory health care services, i.e., physicians and other health care providers. • Nursing and residential care facilities • Pharmacies and drug stores
Neighborhood service facilities	• Senior centers • Arts sites and theaters • Religious organizations • Civic and social organizations • Parks
Food stores/access	• Grocery stores • Special food stores • Fast food restaurants • Drinking places • Coffee shops
Neighborhood deprivation indicators	• Proportion of the population age 16 + who are unemployed • Proportion of families with income below the federal poverty level • Proportion of the population with less than a high school education • Proportion of female-headed families with children • Proportion of Black residents in the neighborhood • Proportion of Hispanic residents in the neighborhood
Neighborhood environmental hazards	• Polluting sites within a census tract plus a half-mile buffer • Highway density: The density of highway stretches, derived through primary and secondary road segments

^a^ Neighborhood measures came from the National Neighborhood Data Archive (2015). All neighborhood measures were expressed as counts per 1,000 population within a census tract.

#### 2.2.3. Covariates.

Demographic covariates included age (in years), gender (male = 0, female = 1), and race/ethnicity (non-Hispanic White = 1, non-Hispanic Black = 2, Hispanic = 3, or other = 4). Socioeconomic characteristics included educational attainment (less than high school = 1, high school or GED = 2, some college = 3, or college or above = 4), household income quartiles (lowest = 1, lower middle = 2, upper middle = 3, highest = 4), and working for pay (no = 0, yes = 1). We also included health-related covariates, including alcohol consumption (not drinking = 0 drinks/day, moderate drinking = men < 3 drinks/day or women < 2 drinks/day, excessive drinking = men ≥ 3 drinks/day or women ≥ 2 drinks/day), smoking status (never smoked = 1, past smoker = 2, or current smoker = 3), and the number of diagnosed chronic conditions (heart disease, lung disease, diabetes, cancer, arthritis, hypertension, and stroke). Disability status was assessed using the Activities of Daily Living (ADL) scale. The scale was constructed by summing binary indicators for difficulty in six basic activities: bathing, eating, dressing, walking across a room, getting in or out of bed, and using the toilet. We also included whether the participant was an apolipoprotein ε4 (APOE ε4) allele carrier. Geographic covariates were urbanicity (urban = 1, suburban = 2, or rural = 3) and census region (Northeast = 1, Midwest = 2, South = 3, or West = 4). Additional control variables, such as marital status, health insurance status, Medicare beneficiary status, and property ownership, were considered but omitted due to lack of statistical significance.

### Statistical analyses

The analysis proceeded in two steps. First, we used Partitioning Around Medoids (PAM), an unsupervised machine-learning algorithm, to identify neighborhood typologies. PAM belongs to the family of partitioning clustering methods that group observations into a set of k clusters. PAM was chosen for its robustness to outliers, its ability to handle heterogeneous data types (numerical and categorical), and its capacity for irregular cluster geometries. This was crucial given the diversity and occasionally large values in our neighborhood measures. Euclidean distance was used as the dissimilarity metric for the clustering procedure. The optimal number of clusters was determined by combining the elbow method, visual inspection, and theoretical interpretability [35]. After obtaining the neighborhood cluster typologies, each cluster was subsequently assigned a descriptive label reflecting the distribution of the mean values of its constituent measures to best characterize its unique features. We then evaluated differences in sample characteristics across neighborhood typologies using the chi-square test or the Kruskal-Wallis test, as appropriate.

Second, multilevel regression models were used to examine the relationship between neighborhood typologies and cognitive function. Given that individuals were nested within census tracts, we adopted a two-level model, with individuals as the first level and census tracts as the second level. We specified census tract-level random intercepts to account for the non-independence of observations within tracts. The covariance structure was set to identity to facilitate model estimation. These models were constructed using a stepwise covariate adjustment approach: Model 1 included neighborhood typologies and adjusted for demographic characteristics and geographic covariates, including age, gender, race, urbanicity, and census region. Model 2 further adjusted for socioeconomic status, including educational attainment, household income, and working status. Model 3 additionally adjusted for chronic conditions, ADL, alcohol consumption, smoking status, and APOE ε4 allele carrier status. All models were weighted using HRS survey weights to account for the complex study design and nonresponse.


$$Y_{ij}=\beta_{\text{0}}+\sum_{l=\text{1}}^{\text{3}}\beta_{l}{Nt}_{l}+\sum \Omega_{p}X_{ij}+u_{j}+e_{ij}\;$$ (1)

where *Y*_*ij*_ represented the cognitive score for individual *i* nested within neighborhood *j*. The key explanatory variable ${Nt}_{l}$was the identified neighborhood typologies. X_*ij*_ represents the covariates included in the model, such as demographic characteristics, geographic covariates, socioeconomic status, and health-related factors. *β*_*0*_ was the intercept. The coefficients of interest are *β*_*l*_, for *l = 1,…,3*, which quantified the relationship between living in different neighborhood typologies and cognitive outcomes, with the last neighborhood typology as the reference group. *Ω*_*p*_ were the coefficients for covariates. u_*j*_ represented the level-2 residual, which measured the deviance between a specific neighborhood’s average cognitive score and the overall average cognitive score for all neighborhoods. e_*ij*_ was the individual-level error term, which was assumed to follow a normal distribution. Statistical tests were two-sided, with a significance level of *p < 0.05*. Data analyses were performed using RStudio version 4.0.2 [36] and STATA 17 [37].

Supplemental analyses were conducted to evaluate the robustness of our findings. First, we assessed the stability of neighborhood typologies by reapplying the PAM clustering approach to more recent NaNDA 2020 data and comparing the resulting typology structure with our primary results. Second, we re-estimated all regression models using multiple imputation by chained equations for missing covariates. Third, we included depression (assessed via the Center for Epidemiological Studies Depression [CES-D] scale) or subjective perceptions of neighborhoods (perceived neighborhood disorder and neighborhood cohesion) in the fully adjusted models to evaluate potential confounding due to their established associations with cognitive function.

## Results

The final analytic sample consisted of 6,480 community-dwelling older adults (Table 2). The mean age was 76 years (standard deviation [SD] = 7.5). A total of 59.1% were female and nearly half (47.0%) had some college education or higher. Over 72.4% of participants were non-Hispanic White, 14.8% were non-Hispanic Black, 10.7% were Hispanic, and 2.2% were from other racial and ethnic groups. Eight percent reported current smoking. Participants had an average of 2.4 chronic conditions (SD = 1.3, range: 0–7). Approximately half of the participants lived in urban areas and 25.8% were APOE ε4 allele carriers. The mean cognitive score for the sample was 14.3 (SD = 4.6, range: 0–27).

**Table 2:** Descriptive statistics of the study sample (Health and Retirement Study, n = 6,480).

Variable	*n (%)/ Mean (SD)* ^*a*^
Cognitive function score (TICS), *mean (SD)*	14.27 (4.60)
Female, *n (%)*	3830 (59.10%)
Race, *n (%)*	
Non-Hispanic White	4688 (72.35%)
Non-Hispanic Black	959 (14.80%)
Hispanic	694 (10.71%)
Other	139 (2.15%)
Age, *mean (SD)*	75.96 (7.45)
Education, *n (%)*	
Less than high school	1149 (17.73%)
High school or GED	2283 (35.23%)
Some college	1536 (23.70%)
College and above	1512 (23.33%)
Working for pay, *n (%)*	
No	5216 (80.49%)
Yes	1264 (19.51%)
Household income quartiles, *n (%)*	
Lowest	1466 (22.62%)
Lower-middle	2002 (30.90%)
Upper-middle	1831 (28.26%)
Highest	1181 (18.23%)
Alcohol consumption, *n (%)*	
Not drinking	4215 (65.05%)
Moderate drinking	1585 (24.46%)
Excessive drinking	680 (10.49%)
Smoking, *n (%)*	
Never smoked	2953 (45.57%)
Past smoker	3025 (46.68%)
Current smoker	502 (7.75%)
Number of chronic conditions, *mean (SD)*	2.43 (1.33)
ADL, *mean (SD)*	0.41 (1.04)
Urbanicity, *n (%)*	
Urban	3255 (50.23%)
Suburban	1435 (22.15%)
Rural	1790 (27.62%)
Census region, *n (%)*	
Northeast	993 (15.32%)
Midwest	1569 (24.21%)
South	2705 (41.74%)
West	1213 (18.72%)
APOE ε4 carrier, *n (%)*	1671 (25.79%)

Abbreviations: TICS = Telephone Interview for Cognitive Status; ADL = Activities of Daily Living; APOE = apolipoprotein E.

^*a*^ Frequency (n) with percentages (%) and means with standard deviations (SD) are presented. These summary statistics are unweighted.

Partitioning Around Medoids (PAM) was performed to identify distinct neighborhood typologies. The optimal number of clusters was primarily determined using the elbow method, which evaluates the reduction in the within-cluster sum of squares (WCSS) across candidate solutions. As shown in S2 Fig, a four-cluster solution was selected at the point where the rate of WCSS decline slowed, indicating diminishing returns with additional clusters. Mean values of individual neighborhood measures for each cluster are presented in S3 Fig and S1 Table. We identified four distinct clusters and labeled each cluster based on its corresponding characteristics. Cluster 1 was labeled as “**low deprivation, green neighborhood**” and was characterized by low neighborhood deprivation, sparse polluting sites, low highway density, very high park density, and a moderate density of neighborhood amenities, such as food stores, social organizations, and healthcare facilities (n = 1,957, 30.2%). Cluster 2 was labeled “**Mid-SES, high hazard neighborhood,**” and featured moderate socioeconomic deprivation, limited access to amenities and green space, and higher levels of environmental hazards (n = 2,451, 37.8%). Cluster 3 was designated as the “**high-amenity neighborhood**” and was characterized by moderate socioeconomic deprivation, but a high concentration of neighborhood amenities, services, and green space (n = 1,068, 16.5%). Finally, Cluster 4 represented the “**disadvantaged neighborhood,**” scoring highest on all deprivation indicators and exhibiting limited access to green space, essential services, and healthcare facilities, yet it was marked by relatively low environmental hazards (n = 1,004, 15.5%).

We present the descriptive statistics of sample characteristics across neighborhood typologies in Table 3. Significant differences were observed across the four types of neighborhood clusters in sociodemographic characteristics, health behaviors, health status, and cognitive scores. Specifically, residents of the low deprivation, green neighborhoods (Cluster 1) were more likely to be older (M_age_ = 76.5, SD = 7.5), non-Hispanic White (87.9%), and college educated (56.9%). They also reported higher household income, with 24.7% belonging to the highest income quartile. This group was less likely to be current smokers (5.8%), had fewer chronic conditions (M = 2.3, SD = 1.3) and was less likely to be APOE ε4 carriers (24.2%). The mean cognitive score for this group was 15.2 (SD = 4.3). In contrast, individuals residing in Cluster 4 presented a profile characterized by multiple disadvantages. They were younger (M_age_ = 74.6, SD = 7.5) and had a lower household income, with only 7.1% in the highest income quartile. Only 28.5% had some college education or higher. Racial and ethnic minority groups were overrepresented in this cluster (e.g., 42.1% non-Hispanic Black; 35.9% Hispanic). Furthermore, they exhibited poorer health behaviors and status, including a higher prevalence of current smoking (11.3%), a higher average number of chronic conditions (M = 2.6, SD = 1.3), and a higher likelihood of being an APOE ε4 carrier (28.3%). They also reported a lower average cognitive score of 12.3 (SD = 4.7). Residents of the remaining two clusters exhibited comparable cognitive performance (M_cluster2_ = 14.4, SD = 4.6 vs. M_cluster3_ = 14.3, SD = 4.5). However, residents of the high-amenity neighborhoods (Cluster 3) demonstrated modestly higher socioeconomic status (e.g., higher educational attainment and household income) yet poorer health behaviors (e.g., higher rates of smoking and excessive alcohol consumption), compared with residents of the Mid-SES, high hazard neighborhoods (Cluster 2).

**Table 3:** Sample characteristics by neighborhood typologies.

	Neighborhood Typologies ^a,b^				*p* ^*c*^
	Cluster 1	Cluster 2	Cluster 3	Cluster 4	
n	1957 (30.20%)	2451 (37.82%)	1068 (16.48%)	1004 (15.49%)	
Cognitive function score (TICS), *mean (SD)*	15.15 (4.28)	14.37 (4.60)	14.28 (4.54)	12.33 (4.67)	<0.001
Female, *n (%)*	1135 (58.00%)	1415 (57.73%)	649 (60.77%)	631 (62.85%)	0.02
Race, *n (%)*					<0.001
Non-Hispanic White	1720 (87.89%)	1994 (81.35%)	779 (72.94%)	195 (19.42%)	
Non-Hispanic Black	100 (5.11%)	269 (10.98%)	167 (15.64%)	423 (42.13%)	
Hispanic	96 (4.91%)	132 (5.39%)	106 (9.93%)	360 (35.86%)	
Other	41 (2.10%)	56 (2.28%)	16 (1.50%)	26 (2.59%)	
Age, *mean (SD)*	76.45 (7.48)	75.96 (7.32)	76.38 (7.51)	74.57 (7.49)	<0.001
Education, *n (%)*					<0.001
Less than high school	183 (9.35%)	374 (15.26%)	176 (16.48%)	416 (41.43%)	
High school or GED	660 (33.73%)	980 (39.98%)	341 (31.93%)	302 (30.08%)	
Some college	497 (25.40%)	570 (23.26%)	274 (25.66%)	195 (19.42%)	
College and above	617 (31.53%)	527 (21.50%)	277 (25.94%)	91 (9.06%)	
Working for pay, *n (%)*					0.63
No	1557 (79.56%)	1983 (80.91%)	860 (80.52%)	816 (81.27%)	
Yes	400 (20.44%)	468 (19.09%)	208 (19.48%)	188 (18.73%)	
Household income quartiles, n (%)					<0.001
Lowest	275 (14.05%)	513 (20.93%)	251 (23.50%)	427 (42.53%)	
Lower-middle	532 (27.18%)	831 (33.90%)	320 (29.96%)	319 (31.77%)	
Upper-middle	667 (34.08%)	704 (28.72%)	273 (25.56%)	187 (18.63%)	
Highest	483 (24.68%)	403 (16.44%)	224 (20.97%)	71 (7.07%)	
Alcohol consumption, *n (%)*					<0.001
Not drinking	1091 (55.75%)	1663 (67.85%)	688 (64.42%)	773 (76.99%)	
Moderate drinking	638 (32.60%)	550 (22.44%)	268 (25.09%)	129 (12.85%)	
Excessive drinking	228 (11.65%)	238 (9.71%)	112 (10.49%)	102 (10.16%)	
Smoking, *n (%)*					<0.001
Never smoked	901 (46.04%)	1100 (44.88%)	490 (45.88%)	462 (46.02%)	
Past smoker	943 (48.19%)	1163 (47.45%)	490 (45.88%)	429 (42.73%)	
Current smoker	113 (5.77%)	188 (7.67%)	88 (8.24%)	113 (11.25%)	
Number of chronic conditions, *mean (SD)*	2.31 (1.31)	2.49 (1.33)	2.38 (1.31)	2.58 (1.32)	<0.001
ADL, *mean (SD)*	0.29 (0.85)	0.39 (1.00)	0.42 (1.05)	0.66 (1.34)	<0.001
Urbanicity, *n (%)*					<0.001
Urban	1206 (61.62%)	893 (36.43%)	560 (52.43%)	596 (59.36%)	
Suburban	481 (24.58%)	510 (20.81%)	169 (15.82%)	275 (27.39%)	
Rural	270 (13.80%)	1048 (42.76%)	339 (31.74%)	133 (13.25%)	
Census region, *n (%)*					<0.001
Northeast	390 (19.93%)	299 (12.20%)	146 (13.67%)	158 (15.74%)	
Midwest	519 (26.52%)	657 (26.81%)	270 (25.28%)	123 (12.25%)	
South	585 (29.89%)	1149 (46.88%)	457 (42.79%)	514 (51.20%)	
West	463 (23.66%)	346 (14.12%)	195 (18.26%)	209 (20.82%)	
APOE ε4 carrier, n (%)	473 (24.17%)	622 (25.38%)	292 (27.34%)	284 (28.29%)	<0.001

Abbreviations: TICS = Telephone Interview for Cognitive Status; ADL = Activities of Daily Living; APOE = apolipoprotein E.

^a^ Frequencies with percentages (%) for categorical variables and means with standard deviations *(SD)* for continuous variables are presented.

^b^ Neighborhood Typologies: Cluster 1: Low deprivation, green neighborhood; Cluster 2: Mid-SES, high hazard neighborhood; Cluster 3: High-amenity neighborhood; Cluster 4: Disadvantaged neighborhood.

^c^ *χ*^*2*^ test and Kruskal-Wallis test were performed to compare statistical differences.

Results from multilevel models are presented in Table 4 (full models are available in S2 Table). Compared with those living in disadvantaged neighborhoods (Cluster 4), residents of low deprivation, green neighborhoods (Cluster 1: *β* = 5.2, *95% CI*: 3.8–6.7), mid-SES, high hazard neighborhoods (Cluster 2: *β* = 5.2, *95% CI*: 1.1–9.4), and high-amenity neighborhoods (Cluster 3: *β* = 5.5, *95% CI*: 2.3–8.7) demonstrated significantly higher cognitive function scores, after adjusting for demographic characteristics and census region (Model 1). Residents of high-amenity neighborhoods (Cluster 3) continued to show significantly higher cognitive function scores (*β* = 4.7, *95% CI*: 1.9–7.4) when socioeconomic characteristics were included (Model 2). The associations were attenuated and became nonsignificant for those in low deprivation, green neighborhoods (Cluster 1: *β* = 1.5, *95% CI*: −0.7–3.8) and those in mid-SES, high hazard neighborhoods (Cluster 2: *β* = 3.0, *95% CI*: −0.5–6.4) (Model 2). In the fully adjusted model (Model 3), residence in high-amenity neighborhoods (Cluster 3) remained significantly associated with higher cognitive function scores, though with some attenuation of the estimate (*β* = 3.9, *95% CI*: 1.2–6.5). Associations for the other neighborhood clusters remained statistically nonsignificant in the fully adjusted model.

**Table 4:** Multilevel regression estimating the association between neighborhood typologies and cognitive function.

	Model 1^a^	Model 2^b^	Model 3^c^
	*β [95% CI]* ^*d*^	*β [95% CI]* ^*d*^	*β [95% CI]* ^*d*^
Neighborhood (ref. Cluster 4: Disadvantaged neighborhood)			
Cluster 1: Low deprivation, green neighborhood	5.24^***^	1.53	1.10
	[3.75, 6.73]	[-0.73, 3.78]	[-1.03, 3.24]
Cluster 2: Mid-SES, high-hazard neighborhood	5.23^*^	2.96	2.53
	[1.06, 9.40]	[-0.51, 6.42]	[-0.71, 5.78]
Cluster 3: High-amenity neighborhood	5.46^**^	4.69^**^	3.85^**^
	[2.25, 8.67]	[1.94, 7.44]	[1.23, 6.47]
Intercept	9.84^**^	22.03^**^	23.14^**^
	[7.70, 11.99]	[18.65, 25.41]	[19.85, 26.43]
Variance (intercept)	17.66	16.05	14.83
	[16.76, 18.62]	[15.21, 16.92]	[14.03, 15.67]
Variance (residual)	7.61	6.03	5.93
	[7.11, 8.16]	[5.64, 6.45]	[5.55, 6.34]

Abbreviations: CI = confidence interval.

^a^ Model 1 adjusted for age, gender, race, urbanicity, and census region.

^b^ Model 2 adjusted for education, income, working status, and Model 1 covariates.

^c^ Model 3 adjusted for comorbidity, Activities of Daily Living (ADL), alcohol consumption, smoking, APOE ε4 carrier status, and Model 2 covariates.

^d^ Regression coefficients and 95% confidence intervals are reported.

** p < 0.01, * p < 0.05.

We conducted supplemental analyses to assess the robustness of our findings. The neighborhood typologies identified using the 2020 NaNDA data exhibited very similar profiles to those identified using the 2015 data (results available upon request). Moreover, the results from the models with multiple imputation were consistent with those of the primary analyses (S3 Table). Additionally, accounting for depression, perceived neighborhood disorder, or perceived neighborhood cohesion resulted in similar findings to those presented in the main analysis (S4 Table).

## Discussion

This study extended the scholarship of the neighborhood exposome by moving beyond individual contextual risk factors to consider the “totality of neighborhood environments.” While some studies have employed composite measures of neighborhood socioeconomic disadvantage or deprivation indices, these indices fail to capture the multifaceted combination of amenities, services, and physical environments that may collectively shape cognitive health in later life. By applying a clustering approach to a large, nationally representative sample of older adults, our analysis yielded two major findings. First, we identified four neighborhood typologies, capturing a spectrum of neighborhoods differentiated by their composite profiles of protective and risk factors. Second, we found that older adults residing in resourceful neighborhoods characterized by extensive facilities and cognitively stimulating services demonstrated better cognitive function compared to those in disadvantaged, resource-scarce neighborhoods.

The identified typologies illustrated the complexity of neighborhood contexts and the way socioeconomic, structural, and environmental factors intersect at the neighborhood level. For instance, while the low-deprivation, green neighborhood cluster (Cluster 1) aligns with established findings that affluent neighborhoods tend to have fewer hazards and greater access to resources [38], we also identified a cluster characterized by moderate socioeconomic deprivation with a high density of amenities and green space (Cluster 3). Social services and facilities are likely to concentrate in areas with a high need for social support among older adults. A recent study found that the density of social care organizations is higher in less affluent neighborhoods with a greater number of older adults [39], which corresponds with the characteristics of Cluster 3 identified in our analysis. At the same time, a neighborhood cluster marked by moderate deprivation and elevated environmental hazards also emerged (Cluster 2), suggesting that residents in these areas may experience overlapping social and environmental risks. Furthermore, Cluster 4 was characterized by pronounced socioeconomic disadvantage coupled with relatively low hazards. Even with fewer toxic exposures, the absence of protective resources, such as healthcare and green space, may still pose long-term health risks for older residents. While research on neighborhood typologies remains scarce and largely exploratory, these patterns resonate with emerging evidence. For example, Humphrey et al. [40] similarly identified four neighborhood clusters within a community-based urban sample, noting that moderate-poverty neighborhoods were characterized by poor or moderate physical environments, whereas wealthy neighborhoods benefited from better physical environments and fewer hazards. Taken together, our identified neighborhood typologies reveal that neighborhoods are complex systems where structural disadvantages and protective resources may coexist in varied combinations, highlighting the importance of a multidimensional framework to capture the health implications of neighborhood environments.

Our findings revealed that older adults residing in high-amenity neighborhoods (Cluster 3) exhibited significantly better cognitive function than those living in neighborhoods characterized by socioeconomic disadvantage and limited access to amenities and resources (Cluster 4). This pattern is consistent with prior cross-national research demonstrating the influence of multidimensional neighborhood characteristics on healthy aging. For example, Wong et al. [41] drawing on data from 33 European countries, found that healthcare services, amenities, and green spaces were important predictors of optimal aging outcomes. Echoing the ecological framework of place [24,42], our findings also highlighted that neighborhoods with greater access to a constellation of amenities, such as healthcare, social services, and civic organizations, were linked to better cognitive function for residents. The cognitive advantages observed in these high-amenity communities may stem from several interconnected mechanisms. Neighborhoods rich in civic and social organizations provide abundant opportunities for cognitively stimulating activities. Access to arts venues, museums, and recreation centers can encourage participation in complex, challenging, and interactive activities that activate multiple cognitive domains and stimulate neuroplasticity [4]. Meanwhile, these settings often encourage physical activity (e.g., group exercise or community volunteering), social interactions, and network building, which in turn support physical health (e.g., cardiovascular health) and reduce stress and inflammation [19,43–45]. Routine engagement in a range ofstimulating experiences may enhance neural efficiency and build cognitive reserve, which in turn buffers against age-related cognitive decline.

Notably, we did not find significant differences in cognitive function between older adults residing in moderate-SES, high hazard neighborhoods (Cluster 2) and those in disadvantaged, resource-scarce neighborhoods (Cluster 4). The nonsignificant finding may reflect the complex interplay of protective and risk factors that operate simultaneously in shaping cognitive health. While a moderate socioeconomic position may be a protective factor, Cluster 2 was characterized by a scarcity of cognitively stimulating resources, such as arts organizations, community centers, and parks, which are established resources supporting healthy aging and cognitive function [10,41]. Importantly, Cluster 2 also exhibited the highest levels of environmental hazards, including high highway density and many polluting sites. These risk factors have been linked to an increased risk of neuroinflammation, accelerated cognitive decline, and the accumulation of Alzheimer’s-related pathologies among older adults [8,17]. Consistent with a prior national cohort study, Finlay et al.[4] found that highway exposure was among the strongest predictors of geographic disparities in cognitive function. Proximity to highways may reduce walkability, heighten safety concerns from fast-moving traffic, increase exposure to air pollution, and limit access to everyday destinations. These factors may further restrict opportunities for physical activity and social engagement. Collectively, these findings align with a growing body of literature suggesting that environmental toxins and hazards may constitute major independent risk factors for late-life cognitive health.

It is noteworthy that cognitive function among older adults in low deprivation, green neighborhoods (Cluster 1) was comparable to that of those in disadvantaged neighborhoods (Cluster 4), despite prior evidence supporting a cognitive benefit from living in socioeconomically advantageous neighborhoods [10,11]. A possible explanation involves residential selection [46]. Our regression analysis showed that residence in low-deprivation, green neighborhoods was significantly associated with better cognitive function in unadjusted models. However, this association was attenuated and became nonsignificance after adjusting for individual-level socioeconomic characteristics and health-related factors. This finding may suggest that the cognitive advantages observed among residents of Cluster 1 might largely be explained by compositional effects. That is, individuals with greater socioeconomic resources and better health profiles may be more likely to reside in affluent communities, rather than reflecting a direct contextual effect of the neighborhood environment itself. Since individuals do not randomly occupy neighborhoods but instead choose or are constrained into residences based on socioeconomic, cultural, and health-related factors, the observed neighborhood effect in the initial model might indicate a systematic sorting process [19,47]. Nonetheless, this finding does not rule out meaningful contextual contributions, as we identified significant associations in the high-amenity neighborhood (Cluster 3) even after accounting for individual-level factors. Therefore, future studies could employ designs capable of addressing selection processes to better capture causal pathways through which neighborhood contextual features shape cognitive health.

Several limitations should be noted. First, while machine-learning clustering methods offer a powerful tool for identifying complex patterns, the approach can introduce uncertainty given the “black box” nature of the algorithm. We chose a four-cluster solution for this analysis based on the elbow method and the distribution of observations across clusters. We also considered a five-cluster solution, but it included a cluster with a small number of observations, which would have resulted in cells with insufficient cases for subsequent analyses. Moreover, because this study relied on a sample from the Health and Retirement Study, replication with a wider range of community and clinical samples is needed to test the external validity and generalizability of the identified neighborhood typologies. Second, while our typologies incorporated key dimensions including socioeconomic status, environmental hazards, and access to amenities and services, other important neighborhood features, such as crime, safety, or transportation infrastructure, were not captured in this analysis. Future research should consider a broader array of neighborhood features to provide a more comprehensive understanding of environmental influences on cognitive aging. Third, given the substantial exclusions due to missing data, the analytic sample was relatively healthier and more socioeconomically advantaged than excluded participants, who may have been at higher risk of cognitive impairment. This selection pattern could lead to an underestimation of neighborhood–cognition associations and limit the generalizability of the findings. Fourth, we were not able to assess changes in neighborhood context and cognition over time due to the cross-sectional design. Our study design precluded causal inference since it was unable to establish temporal precedence between neighborhood characteristics and cognitive function or to rule out reverse causation. Future research employing longitudinal data is crucial to unravel the causal relationship between neighborhood typologies and cognitive health.

## Conclusions

Our study advances understanding of multidimensional neighborhood features in relation to cognitive function among community-dwelling older Americans. We identified four distinct neighborhood typologies characterized by meaningful variation in structural characteristics, amenity access, and environmental conditions. Importantly, our findings underscore significant disparities in cognitive outcomes across neighborhood contexts, with older residents of resource-rich neighborhoods characterized by abundant amenities and cognitively stimulating facilities demonstrating better cognitive function, compared to those in disadvantaged neighborhoods.

These findings should be interpreted as associational, as our cross-sectional study design precludes establishing temporal sequencing between neighborhood features and cognitive outcomes. Accordingly, the results highlight patterns of inequality across neighborhood contexts but do not demonstrate a causal pathway between neighborhood features and differences in cognitive function among U.S. older adults. Nonetheless, our findings establish an important foundation for future longitudinal and experimental research to test whether and through what mechanisms neighborhood typologies influence cognitive aging.

Despite the limitations noted above, our findings have important implications for future research and may help inform the development of place-based hypotheses aimed at promoting cognitive health equity. Our findings suggest that addressing modifiable neighborhood factors could be an important avenue for promoting cognitive health. Neighborhood features related to amenity availability and environmental hazards emerge as potentially modifiable contextual factors that warrant further investigation. While the design and implementation of community-level interventions should be guided by robust longitudinal and experimental evidence, integrating neighborhood assessment into comprehensive geriatric screening and resource allocation may support more equitable approaches to cognitive health promotion among older adults in diverse community settings.

## Supporting information

S1 Fig Sample selection flow chart. (DOCX)

S2 Fig Total within sum of square plot on number of clusters. (DOCX)

S3 Fig The distribution of neighborhood-level measures for neighborhood typologies. (DOCX)

S1 Table Summary of identified neighborhood typologies. (DOCX)

S2 Table Multilevel Regression Estimating the Association between Neighborhood Typologies and Cognitive Function. (DOCX)

S3 Table Multilevel Regression Estimating the Association between Neighborhood Typologies and Cognitive Function with Multiple Imputation. (DOCX)

S4 Table Multilevel Regression Estimating Associations between Neighborhood Typologies and Cognitive Function Adjusting for Additional Covariates. (DOCX)
