# Supplementary material for: Thriving in place: Multidimensional neighborhood typologies and cognitive function among U.S. older adults in the Health and Retirement Study
Source: PLoS One. 2026 Mar 12;21(3):e0344785. doi: 10.1371/journal.pone.0344785 (PMC12981433; doi:10.1371/journal.pone.0344785)
Supplement: S1 Fig — (DOCX) [file pone.0344785.s001.docx]

S1 Figure. Sample selection flow chart

HRS wave 2016

n=20,880

Community-dwelling adults aged 65 and older, n=10,104

Community-dwelling adults aged 65 and older, n=6,827

**Final analytic sample**

**n=6,480**

Excluded participants aged < 65 and proxy respondents,

n=10,776

Excluded participants:

- with incomplete cognition information, n=780
- missing APOE genotype, n=994
- changed residence between 2015 and 2016, n = 827
- missing census tract information, n =676

Excluded participants missing key covariates, n=347
