# Supplementary material for: Thriving in place: Multidimensional neighborhood typologies and cognitive function among U.S. older adults in the Health and Retirement Study
Source: PLoS One. 2026 Mar 12;21(3):e0344785. doi: 10.1371/journal.pone.0344785 (PMC12981433; doi:10.1371/journal.pone.0344785)
Supplement: S2 Fig — (DOCX) [file pone.0344785.s002.docx]

S2 Figure***.*** Total within sum of square plot on number of clusters.

*
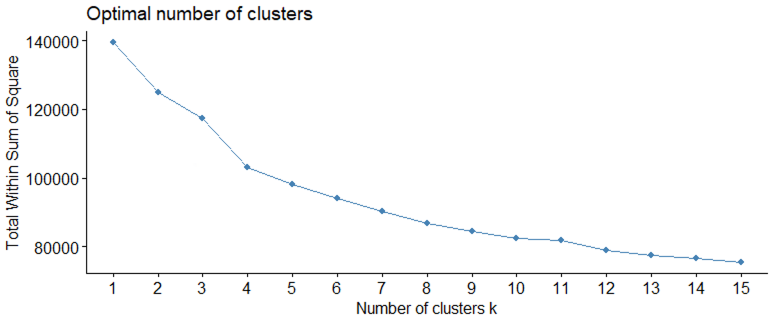
*

The graph shows the within-cluster-sum-of-square (WCSS) values on the y-axis corresponding to the different values of clusters K (on the x-axis). The optimal K value is the point at which the graph forms an elbow or where the rate of WCSS decline slowed, indicating diminishing returns with additional clusters.
