# Supplementary material for: Thriving in place: Multidimensional neighborhood typologies and cognitive function among U.S. older adults in the Health and Retirement Study
Source: PLoS One. 2026 Mar 12;21(3):e0344785. doi: 10.1371/journal.pone.0344785 (PMC12981433; doi:10.1371/journal.pone.0344785)
Supplement: S3 Fig — (DOCX) [file pone.0344785.s003.docx]

S3 Figure. The distribution of neighborhood-level measures for neighborhood typologies


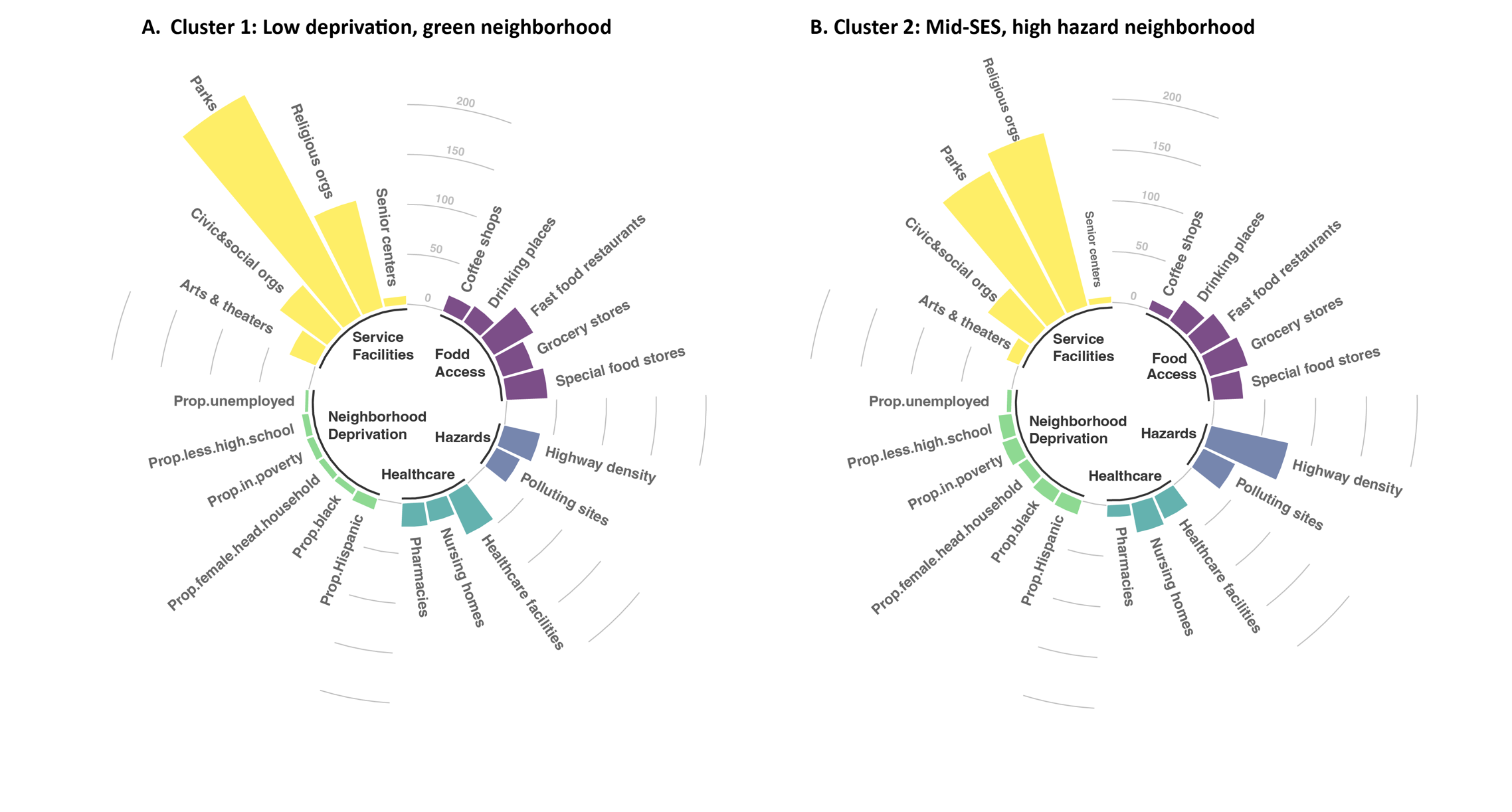


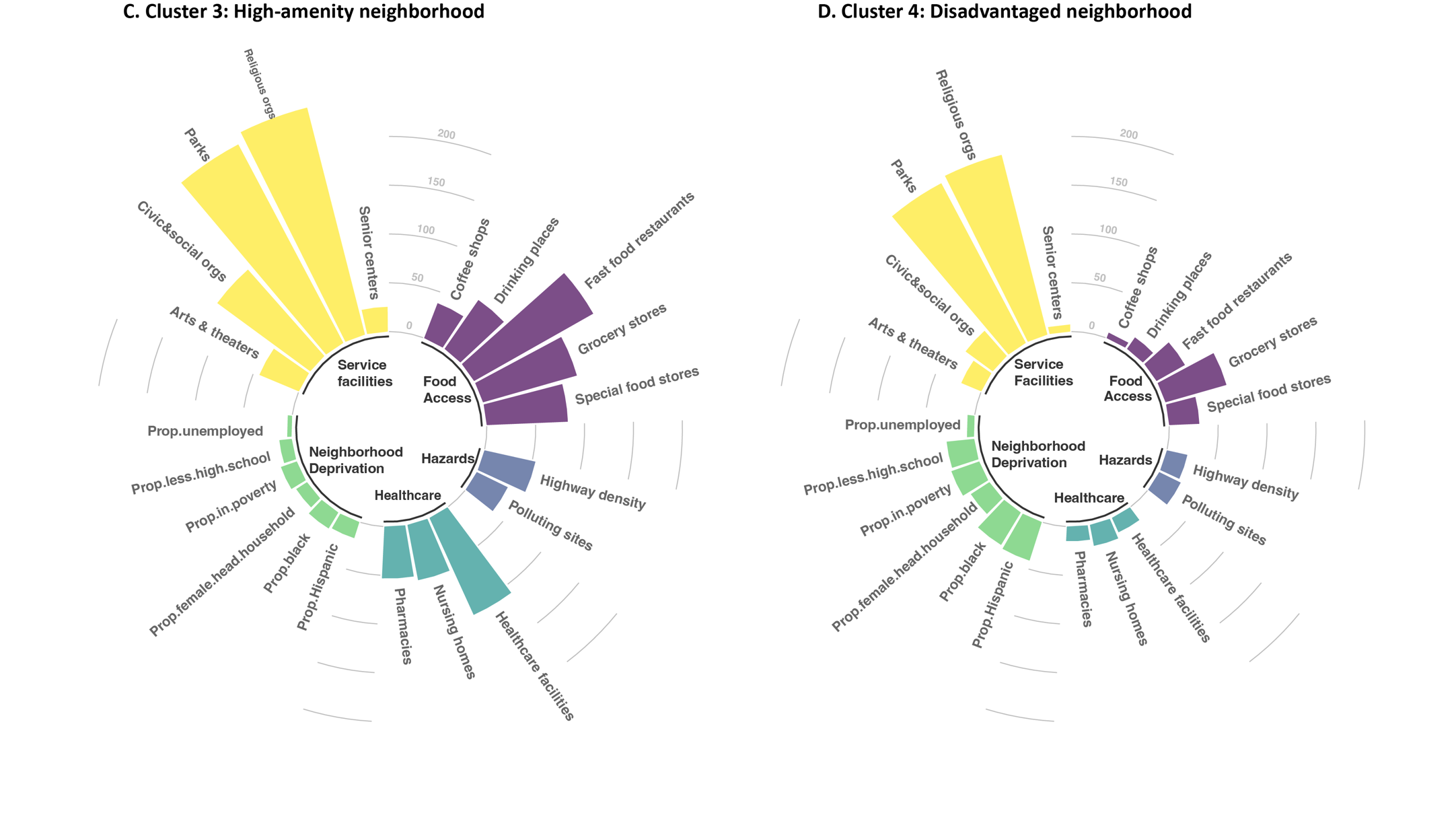


Note: Mean values of individual neighborhood measures for each cluster are presented. All neighborhood measures were in the unit of counts per 1,000 population within a census tract.
