# Supplementary material for: Thriving in place: Multidimensional neighborhood typologies and cognitive function among U.S. older adults in the Health and Retirement Study
Source: PLoS One. 2026 Mar 12;21(3):e0344785. doi: 10.1371/journal.pone.0344785 (PMC12981433; doi:10.1371/journal.pone.0344785)
Supplement: S1 Table — (DOCX) [file pone.0344785.s004.docx]

S1 Table. Summary of identified neighborhood typologies

| Cluster | Label | n (%) | Key Characteristics |
| --- | --- | --- | --- |
| 1 | Low Deprivation, Green Neighborhood | 1957 (30.2%) | Low deprivation, low polluting sites and highway density, high park density, and moderate levels of amenities (food stores, social organizations, healthcare facilities). |
| 2 | Mid-SES, High Hazard Neighborhood | 2451 (38.0%) | Moderate deprivation, limited amenities and green space, and higher levels of hazards. |
| 3 | High-Amenity Neighborhood | 1068 (16.0%) | Moderate deprivation, but highest concentration of neighborhood amenities, facilities, and green space |
| 4 | Disadvantaged Neighborhood | 1004 (15.0%) | Highest deprivation, limited access to green space, services, and healthcare facilities, yet relatively low environmental hazards |
