# Supplementary material for: Thriving in place: Multidimensional neighborhood typologies and cognitive function among U.S. older adults in the Health and Retirement Study
Source: PLoS One. 2026 Mar 12;21(3):e0344785. doi: 10.1371/journal.pone.0344785 (PMC12981433; doi:10.1371/journal.pone.0344785)
Supplement: S2 Table — (DOCX) [file pone.0344785.s005.docx]

S2 Table. Multilevel Regression Estimating the Association between Neighborhood Typologies and Cognitive Function

|  | Model 1 | Model 2 | Model 3 |
| --- | --- | --- | --- |
|  | *β [95% CI] ^a^* | *β [95% CI] ^a^* | *β [95% CI] ^a^* |
| Neighborhood (ref. Cluster 4: Disadvantaged neighborhood) |  |  |  |
| Cluster 1: Low deprivation, green neighborhood | 5.24*** | 1.53 | 1.10 |
|  | [3.75, 6.73] | [-0.73, 3.78] | [-1.03, 3.24] |
| Cluster 2: Mid-SES, high-hazard neighborhood | 5.23* | 2.96 | 2.53 |
|  | [1.06, 9.40] | [-0.51, 6.42] | [-0.71, 5.78] |
| Cluster 3: High-amenity neighborhood | 5.46** | 4.69** | 3.85** |
|  | [2.25, 8.67] | [1.94, 7.44] | [1.23, 6.47] |
| Female |  | 0.89** | 0.91** |
|  |  | [0.62, 1.17] | [0.63, 1.19] |
| Race (ref. Non-Hispanic White) |  |  |  |
| Non-Hispanic Black |  | -2.75** | -2.61** |
|  |  | [-3.67, -1.83] | [-3.53, -1.70] |
| Hispanic |  | -1.96** | -2.13** |
|  |  | [-3.18, -0.73] | [-3.37, -0.88] |
| Other |  | -1.74** | -1.70** |
|  |  | [-2.98, -0.49] | [-2.94, -0.45] |
| Age |  | -0.18** | -0.18** |
|  |  | [-0.20, -0.15] | [-0.20, -0.15] |
| Urbanity (ref. urban) |  |  |  |
| Suburban |  | 2.85 | 2.66 |
|  |  | [-1.89, 7.60] | [-1.54, 6.86] |
| Rural |  | -2.29** | -1.86** |
|  |  | [-3.67, -0.91] | [-3.17, -0.55] |
| Region (ref. Northeast) |  |  |  |
| Midwest |  | 0.46 | 0.44 |
|  |  | [-0.13, 1.05] | [-0.12, 1.00] |
| South |  | -0.02 | -0.01 |
|  |  | [-0.74, 0.69] | [-0.71, 0.68] |
| West |  | 0.30 | 0.32 |
|  |  | [-0.21, 0.81] | [-0.18, 0.83] |
| Education (ref. Less than high school) |  |  |  |
| High School and GED |  | 1.65** | 1.53** |
|  |  | [1.13, 2.18] | [1.00, 2.06] |
| Some college |  | 2.62** | 2.48** |
|  |  | [2.05, 3.19] | [1.91, 3.05] |
| College and above |  | 3.41** | 3.22** |
|  |  | [2.80, 4.03] | [2.61, 3.83] |
| Working for pay |  | 0.92** | 0.79** |
|  |  | [0.53, 1.32] | [0.39, 1.20] |
| Household income quantiles (ref. lowest) |  |  |  |
| Lower-middle |  | 1.08** | 1.00** |
|  |  | [0.55, 1.60] | [0.48, 1.52] |
| Upper-middle |  | 1.05** | 0.94** |
|  |  | [0.46, 1.64] | [0.36, 1.53] |
| Highest |  | 1.41** | 1.20** |
|  |  | [0.70, 2.12] | [0.50, 1.90] |
| Alcohol consumptions (ref. not drinking) |  |  |  |
| Moderate drinking |  |  | 0.43* |
|  |  |  | [0.04, 0.82] |
| Excessive drinking |  |  | -0.10 |
|  |  |  | [-0.61, 0.41] |
| Smoking (ref. never smoked) |  |  |  |
| Past smokers |  |  | -0.02 |
|  |  |  | [-0.34, 0.30] |
| Current smoker |  |  | -0.70* |
|  |  |  | [-1.27, -0.13] |
| Number of chronic conditions |  |  | -0.10 |
|  |  |  | [-0.23, 0.02] |
| ADL |  |  | -0.36** |
|  |  |  | [-0.56, -0.16] |
| APOE ε4 carrier |  |  | -0.40* |
|  |  |  | [-0.75, -0.05] |
| Intercept | 9.84** | 22.03** | 23.14** |
|  | [7.70, 11.99] | [18.65, 25.41] | [19.85, 26.43] |
| Variance (intercept) | 17.66 | 16.05 | 14.83 |
|  | [16.76, 18.62] | [15.21, 16.92] | [14.03, 15.67] |
| Variance (residual) | 7.61 | 6.03 | 5.93 |
|  | [7.11, 8.16] | [5.64, 6.45] | [5.55, 6.34] |

Abbreviations: CI = confidence interval; ADL = Activity of Daily Living; APOE = apolipoprotein E.

^a^ Coefficients and 95% confidence intervals were reported.

*** p<0.01, * p<0.05*
