# Supplementary material for: Thriving in place: Multidimensional neighborhood typologies and cognitive function among U.S. older adults in the Health and Retirement Study
Source: PLoS One. 2026 Mar 12;21(3):e0344785. doi: 10.1371/journal.pone.0344785 (PMC12981433; doi:10.1371/journal.pone.0344785)
Supplement: S3 Table — (DOCX) [file pone.0344785.s006.docx]

S3 Table. Multilevel Regression Estimating the Association between Neighborhood Typologies and Cognitive Function with Multiple Imputation

|  | Cognitive function ^a^ |
| --- | --- |
|  | *β [95% CI]* |
| Neighborhood (ref. Cluster 4: Disadvantaged neighborhood) |  |
| Cluster 1: Low deprivation, green neighborhood | 1.08 [-0.47, 2.64] |
| Cluster 2: Mid-SES, high-hazard neighborhood | 2.20 [-0.56, 4.97] |
| Cluster 3: High-amenity neighborhood | 4.65^***^ [2.16, 7.13] |
| Intercept | 25.90^**^ [21.80, 29.99] |
| Variance (intercept) | 15.79 [14.95, 16.67] |
| Variance (residual) | 5.77 [5.38, 6.19] |

Abbreviations: CI = confidence interval

^a^  Regression coefficients and 95% confidence intervals were reported, adjusting for age, gender, race, urbanity, census region, education, income, working status, comorbidity, Activity of Daily Living (ADL), alcohol consumption, smoking, APOE ε4 carrier status.

*** p<0.01, * p<0.05.*
