# Supplementary material for: Thriving in place: Multidimensional neighborhood typologies and cognitive function among U.S. older adults in the Health and Retirement Study
Source: PLoS One. 2026 Mar 12;21(3):e0344785. doi: 10.1371/journal.pone.0344785 (PMC12981433; doi:10.1371/journal.pone.0344785)
Supplement: S4 Table — (DOCX) [file pone.0344785.s007.docx]

S4 Table. Multilevel Regression Estimating Associations between Neighborhood Typologies and Cognitive Function Adjusting for Additional Covariates ^a^

|  | Cognitive Function (*β,* 95% CI) ^a^ | | |
| --- | --- | --- | --- |
|  | Adjusting for covariates and depression | Adjusting for covariates and neighborhood disorder | Adjusting for covariates and neighborhood cohesion |
| Neighborhood (ref. Cluster 4: Disadvantaged neighborhood) |  |  |  |
| Cluster 1: Low deprivation, green neighborhood | 1.12 | 1.27 | 1.78 |
|  | [-1.03, 3.27] | [-1.74, 4.28] | [-1.24, 4.80] |
| Cluster 2: Mid-SES, high-hazard neighborhood | 2.54 | 2.77 | 3.24 |
|  | [-0.79, 5.87] | [-0.85, 6.40] | [-0.51, 6.99] |
| Cluster 3: High-amenity neighborhood | 3.77** | 4.88** | 5.21** |
|  | [1.16, 6.38] | [1.30, 8.46] | [1.62, 8.82] |
| Intercept | 23.53** | 23.20** | 21.29** |
|  | [20.10, 26.86] | [18.75, 27.65] | [16.79, 25.78] |
| Variance (intercept) | 15.32 | 15.84 | 15.83 |
|  | [14.50, 16.18] | [14.94, 16.80] | [14.93, 16.79] |
| Variance (residual) | 5.89 | 5.45 | 5.46 |
|  | [5.51, 6.30] | [5.05, 5.87] | [5.06, 5.88] |

Abbreviations: CI = confidence interval

^a^ Regression coefficients and 95% confidence intervals were reported. All models adjusted for age, gender, race, urbanity, census region, education, income, working status, comorbidity, Activity of Daily Living (ADL), alcohol consumption, smoking, APOE ε4 carrier.

*** p<0.01, * p<0.05.*
